# Ross Procedure in Children: 17‐Year Experience at a Single Institution

**DOI:** 10.1161/JAHA.113.000153

**Published:** 2013-04-24

**Authors:** Sharman P. Tan Tanny, Matthew S. Yong, Yves d'Udekem, Remi Kowalski, Gavin Wheaton, Luigi D'Orsogna, John C. Galati, Christian P. Brizard, Igor E. Konstantinov

**Affiliations:** 1 Royal Children's Hospital, Melbourne, Australia (S.P.T.T., M.S.Y., Y.U., R.K., C.P.B., I.E.K.); 2 University of Melbourne, Australia (S.P.T.T., M.S.Y., Y.U., R.K., C.P.B., I.E.K.); 3 Murdoch Children's Research Institute, Melbourne, Australia (Y.U., R.K., J.C.G., C.P.B., I.E.K.); 4 Women's and Children's Hospital, Adelaide, Australia (G.W.); 5 Princess Margaret Hospital for Children, Perth, Australia (L.O.); 6 Department of Mathematics and Statistics, La Trobe University, Melbourne, Australia (J.C.G.)

**Keywords:** aorta, pediatrics, surgery, survival, valves

## Abstract

**Background:**

The Ross procedure in children carries substantial mortality and reoperation rate. Aortic root dilatation is of concern. To prevent dilatation of the neoaortic root, but permit normal growth, we began to apply an absorbable poly‐(*p*‐dioxanone)‐filaments (PDS) band at the sino‐tubular (ST)‐junction.

**Methods and Results:**

All children (n=100) who underwent Ross procedure during 1995–2012 were studied. Mean age at operation was 8.6±6.1 years (median 8.3 years, range 3 days to 18 years); 19 patients were younger than 1 year of age. The root replacement (n=91, Ross‐Konno procedure in 29 patients), root inclusion (n=6), and subcoronary implantation (n=3) techniques were used. Operative mortality was 6% (6/100, 4 neonates, 2 infants). Age of <1‐year at time of operation was a risk factor for early death (*P*<0.001). Mean follow‐up time was 7.0±4.8 years (median 7.4 years, range 5 days to 16 years). Late mortality was 4.3% (4/94). Freedom from moderate or greater neoaortic valve insufficiency (AI) at 5 and 10 years was 89% and 83%, respectively. Freedom from neoaortic valve reoperation at 5 and 10 years was 96% and 86%, respectively. Aortic dilatation to Z‐score >4 was greatest at the ST‐junction (23%, 11/48) compared to the aortic annulus (17%, 11/66) and sinuses (14%, 7/50). Since 2001, a PDS band was placed around the ST‐junction in 19 patients. Survivors with the PDS band had less AI (0 versus 20%, *P*=0.043) compared to survivors (n=35) without the PDS at 4.1±3 years.

**Conclusions:**

The Ross procedure in children can be performed with acceptable results. Children younger than 1 year of age have higher mortality, but not an increased autograft reoperation rate. Stabilization of the ST‐junction may reduce AI.

## Introduction

Replacement of the aortic valve with a pulmonary autograft (Ross procedure) was introduced in the late 1960s,^1^ and good results have been reported in both adults^2–4^ and children.^5–10^ However, younger age at the time of the Ross procedure appears to be a risk factor for mortality and reoperation.^11–12^ Progressive neoaortic valve insufficiency (AI) and the need to replace the right ventricular outflow tract (RVOT) conduit in a growing child are key issues.^13^ The main concern in children is the dilatation of the neoaortic root, which leads to progression of AI.^14–15^ While stabilization of the aortic root can be achieved in most adult patients,^16–17^ prevention of aortic root dilatation in a growing child can be problematic. To reduce aortic root dilatation and the rate of reoperations on the neoaortic root, we began to apply an absorbable poly‐(*p*‐dioxanone)‐filaments (PDS) band to stabilize the sino‐tubular (ST)‐junction. Herein we describe our results with the Ross procedure and midterm outcomes of the ST‐junction stabilization technique.

## Patients and Methods

### Patients

This study was approved by the Human Research Ethics Committee at the Royal Children's Hospital (RCH). All patients who underwent a Ross procedure at the RCH between November 1995 and April 2012 were included in this retrospective study. Medical records were reviewed. Two researchers (S.P.T.T. and R.K.) reviewed all echocardiograms and recorded the diameters of the pulmonary and aortic annulus along with preoperative aortic insufficiency and stenosis. Follow‐up echocardiogram reports and letters were obtained from the patients' respective cardiologists. At last follow‐up, postoperative autograft measurements and Z‐scores^18^ were obtained at the annulus, ST‐junction, and sinus of valsalva. Early death was defined as death occurring within 30 days of the Ross procedure having been performed or before discharge from the hospital.

Patient characteristics are outlined in Table 1. There were 63 male patients and the mean age at operation was 8.6±6.1 years (range 3 days to 18 years). There were 6 neonates (≤30 days of age), 13 infants, and 81 children older than 1 year of age.

**Table 1.** Patient Characteristics

Characteristic	% or Mean±SD (range) For n=100 Patients
Male	63%
Age at operation, y	8.6±6.1 (3 days to 18 years)
<1 year	19%
1 to 10 years	36%
11 to 18 years	45%
Diagnosis	
Endocarditis	10%
Isolated aortic stenosis	20%
Isolated aortic insufficiency	18%
Combined aortic stenosis and insufficiency	38%
Subaortic stenosis	14%
Aortic valve morphology	
Unicuspid	3%
Bicuspid	40%
Tricuspid	57%
Associated diagnosis	
Rheumatic fever	2%
Interrupted aortic arch	4%
Ventricular septal defect	6%
Atrial septal defect	1%
Coarctation of the aorta	10%
Endomyocardial fibroelastosis	3%
Cor triatriatum	1%
Prior procedures	
Balloon valvotomy only	20%
Surgical valve repair only	33%
Surgical valve repair and balloon valvotomy	11%

### Operative Techniques

All patients underwent surgery via sternotomy with standard cardiopulmonary bypass (CPB), hypothermia, and antegrade cardioplegia. Median weight at operation was 25.1 kg (range 12.7 to 49 kg) with a mean body surface area of 1.0±0.54 m^2^ (range 0.17 to 2.3 m^2^). The median length of CPB was 215 minutes (IQR 178 to 253 minutes). Median aortic cross‐clamp time was 149 minutes (IQR 122 to 182 minutes). Circulatory arrest during operation was utilized for only 1 patient for 12 minutes. The median lowest temperature was 28°C (IQR 28 to 32°C). The root replacement (n=91, Ross‐Konno procedure in 29 patients), root inclusion (n=6), and subcoronary implantation (n=3) techniques were used. The autograft was sutured to the left ventricular outflow tract using a running prolene suture. Right ventricular to pulmonary artery (RV‐to‐PA) continuity was established using a homograft (aortic or pulmonary) or a Contegra (Medtronic Inc.) conduit in all the patients. The median conduit size used was 20 mm (IQR 18 to 22 mm). Since 2001, 19 patients had PDS banding around the ST‐junction with the anticipation of reducing progression of AI.

Concomitant procedures were performed in 21 patients, including 1 or more of: mitral valve repair (n=9), aortic arch repair (n=4), subaortic membrane resection (n=4), patent foramen ovale closure (n=2), patent ductus arteriosus ligation (n=2), mitral valve replacement (n=1), left atrial aneurysm repair (n=1), endocardial fibroelastosis resection (n=1), ventral septal defect closure (n=1), relief of subaortic stenosis (n=1), myomectomy (n=1), ascending aorta enlargement (n=1), and atrial septal defect closure (n=1). The repair was assessed intraoperatively with transoesophageal echocardiography. The median length of stay in intensive care unit was 25 hours (IQR 21 to 67 hours) and the median length of hospital stay was 7 days (IQR 6 to 12 days).

### Statistical Analysis

All data were analyzed with Stata Statistical Software: Release 10 (StataCorp LP). Descriptive statistics for continuous variables were expressed as mean±standard deviation (range), whereas skewed continuous data are presented as medians (interquartile range). A Student's *t* test was used to compare continuous variables. Categorical variables were summarized as frequencies and percentages. A Fisher's exact test was used to compare categorical or binary variables.

Kaplan–Meier actuarial survival curves were used to analyze and plot time‐related endpoints. Association of age at Ross procedure less than 1 year with early mortality and comparison of aortic insufficiency between PDS and control groups was assessed using a chi‐squared test and confirmed using a Fisher's exact test. Cox proportional hazards regression analysis was used to identify risk factors for time‐related outcomes. Variables tested in the univariate analysis included weight, age, syndrome, isolated aortic stenosis (unicuspid, bicuspid, tricuspid), isolated aortic insufficiency, combined aortic stenosis and insufficiency, endocarditis, rheumatic heart disease, root replacement technique, root inclusion technique, subcoronary technique, Ross‐Konno procedure, PDS use, concomitant surgery, bypass time, and cross‐clamp time. Of the endpoints examined, due to the number of outcomes, multivariable risk analysis was feasible only for any reoperation. For this endpoint, the number of risk factors included in the model was limited to the 3 most likely predictors from the univariate analysis (due to the limited number of reoperations in total).

## Results

### Survival

**Figure 1.**
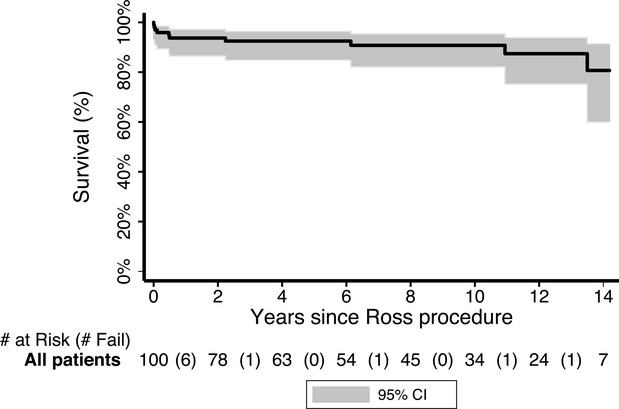
Overall survival.

The causes of death are summarized in Table 2. Early mortality was 6% (6/100), while late mortality was 4.3% (4/94), occurring at 2, 6, 11, and 14 years after the Ross procedure. Overall mortality for the Ross procedure was 10% (10/100). Univariate analysis for overall death (Table 3) identified younger age at operation (*P*=0.016), lower weight at operation (*P*=0.018), longer CPB time (*P*<0.0001), and longer aortic cross clamp time (*P*<0.0001) as characteristics associated with increased mortality. The small number of deaths prevented any conclusions to be drawn from a multivariate analysis. Kaplan–Meier estimates of overall survival (Figure 1) at 5 years were 92.5% (95% CI: 84.8 to 96.4) and at 10 years were 90.8% (95% CI: 82.1 to 95.3).

**Table 2.** Description of Deaths

Patient	Description	Prior surgery	Operation (Year)	Concomitant	Postoperative ECMO	Death (Year)	Cause of Death
1	2.5 kg neonate, late diagnosis of severe heart failure	Nil	Ross‐Konno (2001)	Aortic arch repair	No	Early (2001)	Failure to wean off bypass. Parents declined ECMO
2	3.6 kg neonate with endocardial fibroelastosis	Balloon aortic valvotomy	Ross‐Konno (2003)	Mitral valve repair	No	Early (2003)	Sepsis and multiorgan failure
3	11 kg infant with endocarditis, aortic root abscess, preoperative cardiac arrest	Nil	Ross (2003)	Nil	No	Early (2003)	Stroke
4	2.7 kg neonate with endocarditis, aortic root abscess	Nil	Ross (2008)	Mitral valve repair	Yes	Early (2008)	Sepsis and multiorgan failure
5	4.4 kg infant with Shone complex	Mitral valve repair	Ross‐Konno (2010)	Mitral valve replacement	Yes	Early (2011)	Low cardiac output
6	3.3 kg neonate with endocardial fibroelastosis.	Surgical aortic valvotomy, LVAD and ECMO	Ross‐Konno (2010)	Aortic arch repair and resection of fibroelastosis	Yes	Early (2010)	Stroke
7	5‐year‐old child with endocarditis	Resection of subaortic stenosis, surgical aortic valvotomy	Ross (1996)	Nil	No	Late (2002)	Noncardiac related, accidental trauma
8	11‐year‐old child with Shone complex	Coarctation repair, balloon aortic valvuloplasty, LVOT reconstruction, resection of subaortic stenosis	Ross‐Konno (1996)	Aortic arch repair	No	Late (2007)	Stroke at reoperation on aortic arch and RV‐to‐PA conduit replacement
9	3‐year‐old child with endocardial fibroelastosis.	Balloon aortic valvotomy	Ross (1997)	Nil	No	Late (2011)	Congestive cardiac failure with total artificial heart replacement after RV–PA conduit and aortic root replacement
10	12‐year‐old child with rheumatic heart disease	Mitral valve repair	Ross (2004)	Mitral valve repair	No	Late (2006)	Sudden unexpected death

ECMO indicates extracorporeal membrane oxygenation; LVAD, left ventricular assist device; LVOT, left ventricular outflow tract; RV‐to‐PA, right ventricle to pulmonary artery.

**Table 3.** Risk Factors for Overall Mortality by Univariable Analysis

Variable	HR	*P*‐Value	95% CI
Cardiopulmonary bypass time, min	1.02	<0.0001	1.01 to 1.03
Aortic cross clamp time, min	1.02	<0.0001	1.01 to 1.04
Age at Ross procedure, y	0.83	0.016	0.71 to 0.96
Weight at Ross procedure, kg	0.93	0.018	0.87 to 0.99

HR indicates hazard ratio for a 1 unit change in the given factor.

### Reoperation

**Figure 2.**
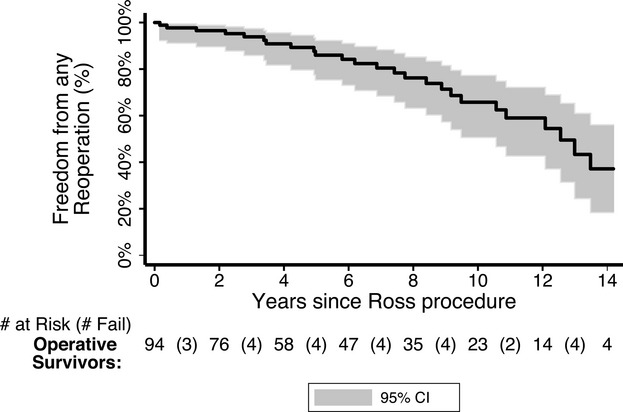
Freedom from any reoperation.

Reoperation was required in 27 patients (27.3%, 27/94; 6 patients did not survive to hospital discharge after the Ross procedure and were therefore excluded from the reoperation analysis). Freedom from any reoperation (Figure 2) at 5 and 10 years was 86% (95% CI: 75.3 to 92.3) and 65.8% (95% CI: 50.6 to 77.3), respectively. On univariate Cox regression analysis, homograft size (*P*<0.0001; hazard ratio: 0.76 for each 1 mm increase in size; 95% CI: 0.67 to 0.86), younger age at operation (*P*=0.001; hazard ratio: 0.88 for each 1 year increase in age; 95% CI: 0.81 to 0.95), and lower weight at operation (*P*=0.032; hazard ratio: 0.98 for each 1 kg increase in weight; 95% CI: 0.95 to 1.0) were identified as risk factors. However, only homograft size (*P*=0.012; hazard ratio: 0.78 for each 1 mm increase in size; 95% CI: 0.64 to 0.95) was statistically significant on multivariable analysis.

**Figure 3.**
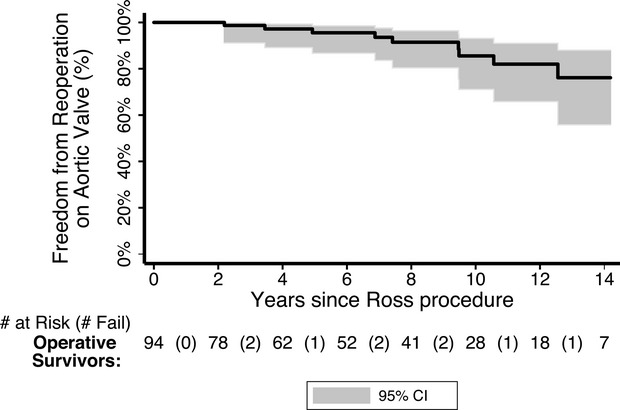
Freedom from autograft reoperation.

Autograft reoperation was required in 9 (9.1%, 9/99) patients with neoaortic root dilatation leading to AI. Mean length of time to aortic reoperation after Ross procedure was 7.4±3.4 years (2.2 to 12.6 years). Five patients had an aortic valve or root replacement, 1 patient had ascending aortic arch patch repair, and 3 patients had an aortic valve repair. The prostheses used included a 23 mm On‐X aortic valve (On‐X Life Technologies, Inc.) (n=2), an ATS Advanced Performance 22 mm valve (ATS Medical, Inc.) (n=1), a 25 mm St Jude aortic valve (St Jude Medical, Inc.) (n=1), and a 26 mm valsalva Dacron graft (n=1). Of the 3 patients who underwent aortic valve repairs, 1 patient eventually had an aortic valve replacement with a 23 mm On‐X aortic valve (On‐X Life Technologies, Inc.). Freedom from neoaortic valve reoperation (Figure 3) at 5 and 10 years was 95.5% (95% CI: 86.7 to 98.5) and 85.6% (95% CI: 71.1 to 93.1), respectively. There were no risk factors identified on univariate Cox regression analysis.

**Figure 4.**
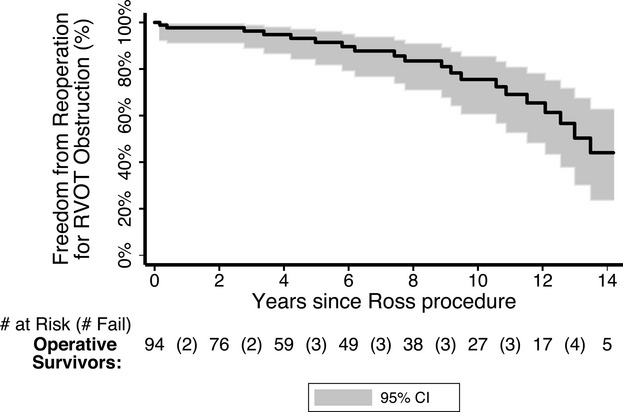
Freedom from right ventricular outflow tract (RVOT) reoperation.

Replacement of the RVOT conduit was required in 20 (20.2%, 20/99) patients at a mean duration of 7.7±4.1 years (2 months to 13.5 years) after the Ross procedure. Freedom from RVOT reoperation (Figure 4) at 5 years is 91.4% (95% CI: 81.7 to 96.1) and at 10 years is 75.5% (95% CI: 60.6 to 85.4). Risk factors on univariate Cox regression analysis included homograft size (*P*<0.0001; hazard ratio: 0.7 for each 1 mm increase in size; 95% CI: 0.59 to 0.82), age at Ross procedure (*P*<0.0001; hazard ratio: 0.81 for each 1 year increase in age; 95% CI: 0.72 to 0.91), and weight at Ross procedure (*P*=0.017; hazard ratio: 0.96 for each 1 kg increase in weight; 95% CI: 0.94 to 0.99).

Other reoperations and reinterventions included mitral valve replacement (n=4), heart transplant (n=1), coronary artery surgery (n=1), and intervention with balloon dilatation (n=5). Four (5%, 4/85) patients required permanent pacemaker implantation after Ross procedure.

### Autograft Competency

**Figure 5.**
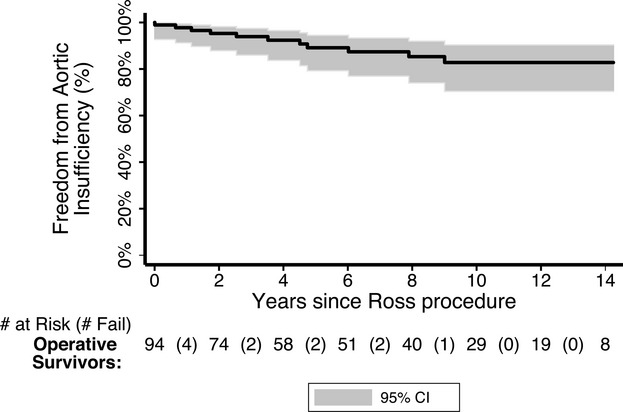
Freedom from greater than moderate aortic insufficiency.

Among hospital survivors, there were 11 (11.7%, 11/94) patients who developed moderate to severe AI. The Kaplan–Meier estimate of freedom from greater‐than‐moderate AI is outlined in Figure 5. Five patients with moderate to severe AI after Ross procedure underwent reoperation on the aortic valve with good outcomes (3 patients having no AI and 2 patients having trivial AI). At last follow‐up, trivial AI was found in 23 patients (25.6%, 23/90), mild AI was found in 25 patients (27.8%, 25/90), moderate or severe AI was found in 6 patients (6.7%, 6/90), and the remaining 36 patients (40%, 36/90) had no AI. Post Ross procedure, aortic dilatation of Z‐score >4 was greatest at the ST‐junction (23%, 11/48) when compared to the aortic annulus (17%, 11/66) and sinuses (14%, 7/50). Only 6.7% (3/45) had a Z‐score >4 in all 3 characteristics. One patient had aortic stenosis.

**Figure 6.**
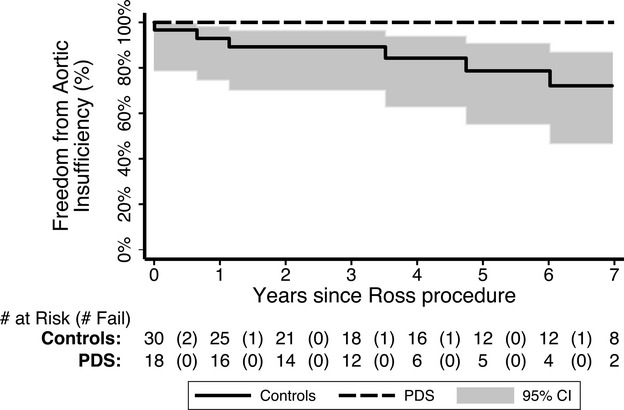
Freedom from greater than moderate aortic insufficiency, comparison between poly‐(*p*‐dioxanone)‐filaments (PDS) band group and control group.

At our institution, a PDS band was used in 19 patients. We compared them with a control group of 35 patients who underwent the Ross procedure without use of a PDS band (Table 4). These patients were then evaluated based on age, weight, year of surgery, and length of follow‐up. There were no differences between operative mortality (*P*=0.31), late mortality (*P*=0.19), and neoaortic reoperation (*P*=0.45). However, there was a reduction in moderate or greater AI (*P*=0.043) in the PDS group (Table 4). Kaplan–Meier comparison of AI in PDS versus non‐PDS group is shown in Figure 6.

**Table 4.** Comparison Between the PDS Band Group and Control Group

	Overall (n=54)	PDS Group (n=19)	Control Group (n=35)	*P*‐Value
Age at surgery, years (range)	8.2±6.2 (0 to 18)	8.5±5.1 (0.2 to 14.9)	8.1±6.9 (0 to 18)	0.81
Weight, kg (range)	31.2±23.5 (2.5 to 84)	31.5±21.3 (4.0 to 76.2)	31.0±25 (2.5 to 84)	0.95
BSA, m^2^ (range)	1.0±0.6 (0.2 to 2.0)	1.0±0.5 (0.2 to 1.9)	1.0±0.6 (0.2 to 2.0)	0.67
Follow‐up time, years (range)	4.1±3.0 (0.2 months to 9.2 years)	3.7±2.5 (0.8 months to 9.2 years)	4.3±3.2 (0.2 months to 9.1 years)	0.47
Aortic reoperation, %	1 (1.9)	0 (0)	1 (2.9)	0.45
Homograft reoperation, %	5 (9.4)	2 (10.5)	3 (8.8)	0.84
Early death, %	6 (11.1)	1 (5.3)	5 (14.3)	0.31
Late death, %	1 (2.1)	1 (5.6)	0 (0)	0.19
Moderate or greater aortic insufficiency, %	6 (12.5)	0 (0)	6 (20)	0.043
Sinotubular junction Z‐score>4, %	4/23 (17.4)	2/7 (28.6)	2/16 (12.5)	0.56

PDS indicates poly‐(*p*‐dioxanone)‐filaments; BSA, body surface area.

### Follow‐Up

Mean follow‐up for the 90 survivors was 7.3±4.8 years (1 month to 16 years). Concurrent follow‐up (within the last 3 years) was obtained for 92.2% (83/90) of the patients, and the remainder were lost to follow‐up. Of the survivors, 96.7% (87/90) of the patients were in New York Heart Association (NYHA) class 1 and 3.3% (3/90) were in class 2.

## Discussion

Although the Ross procedure was initially intended for the treatment of aortic valve disease in adults,^1^ it is currently utilized in the pediatric population as well. Herein we reported a 17‐year experience at a single institution using the Ross procedure in children.

An operative mortality rate of 6% was observed in the present study. This is consistent with other studies in children where operative mortality ranged from 0% to 16%.^5,7–9,15,19–21^ Takkenberg et al^12^ reviewed the literature regarding the outcomes of the Ross procedure in children and adults and their meta‐analysis found, on average, an increased rate of early mortality in children (4.2%) compared to adults (3.2%).^12^ In our study, patients younger than 1 year were at a higher risk of early mortality (32%, 6/19). All of the early deaths occurred in the neonatal and infant age group. The patients who died presented in a critical state and necessitated urgent surgery. This is similar to the results reported by Alsoufi et al^11^ (n=151, median age of 8.6 years at operation) who identified age younger than 1 year as a predictor of early mortality. In addition, Kadner et al^8^ (n=52, median age of 5 years at operation) observed a high early mortality in neonates and infants who presented in poor preoperative condition. Overall, a 10‐year survival rate of 91% in our study is comparable to the 90% reported by Horer et al^9^ (n=152, median age of 10.1 years at operation). In our study, it appeared that younger and smaller children had worse outcomes, especially those who had preoperative endocarditis.

In this present study, the pulmonary autograft performed well with good intermediate‐term durability. Autograft failure leading to AI was the cause of reoperation in all 9 patients. We report a 10‐year rate of freedom from aortic valve or root reoperation of 86%, comparable to the 10‐year rate reported by Alfousi et al^15^ of 79%. The meta‐analysis by Takkenberg et al^12^ reported significant variability between studies for autograft deterioration. They suggested that differences in surgical technique might account for some of these variations. Horer et al^9^ (mean follow‐up of 6.1 years) and Shinkawa et al^5^ (median follow‐up of 6 years) reported a rate of freedom from autograft reoperations at 10 years of 96% and 95.2%, respectively, demonstrating that excellent results may be achieved in the pediatric population. Notably, Shinkawa et al^5^ reported a rate of freedom from autograft reoperation at 15 years to be 63.5%, suggesting a greater proportion of patients do require future reoperations when the follow‐up time is increased. Similarly, Horer et al^9^ identified longer follow‐up time as a significant risk factor for autograft failure, suggesting that the number of autograft reoperations may increase after the first decade.

Increase in aortic root Z‐scores with time, suggesting progressive autograft dilatation, is a key concern with the Ross procedure, and has been reported by several authors.^16,22–23^ The challenge faced in the pediatric population is the potential for AI to develop as the neoaortic root increases in size becoming out of proportion to the somatic growth of the child or young adult.^24^ Inclusion, sub‐coronary, and reinforcement techniques to improve the durability of the autograft have been utilized in adult populations and mid‐term results have been promising.^16–17,16–27^ However, stabilization of the neoaortic root that would, at the same time, permit somatic growth is challenging in children. In our study, 40% of patients did not have any AI, 53% had trivial to mild AI and 7% had moderate to severe AI. Consistent estimation of neoaortic root dimensions has been more difficult as most cardiologists, particularly those involved in adult outpatient care, did not record all root dimensions. Nonetheless, from the data available to us, it appears that the most significant root dilatation occurs at the ST‐junction. Thus, since 2001, a PDS band was placed around the ST‐junction in some patients in an attempt to prevent root dilatation at this level and, hopefully, to minimize subsequent AI. According to the manufacturer's specification, approximately 35% of the PDS band's original strength remains 21 days after implantation. The PDS band subsequently loses all original strength within approximately 50 days and is absorbed after roughly 6 months. This would, theoretically, allow scar formation around the ST‐junction to stabilize it while allowing subsequent normal growth. The patients were not randomized prospectively to PDS band application, but the PDS band was applied by 2 surgeons as a personal preference. On analysis of our results, the technique did not significantly decrease the rate of autograft reoperation at mid‐term follow‐up. However, AI was reduced significantly in the PDS group. These results appear promising and further study of the PDS band technique is warranted in order to observe whether the reduction in AI translates into fewer autograft reoperations in the PDS group over time.

A weak point of the Ross procedure is the potential for an isolated aortic valve disease to be transformed into a double valve disease. The most common indication for reoperation in this cohort was for replacement of RV‐to‐PA conduit. As the patients grow, reoperation to replace the conduit can be expected. In our study, freedom from RV‐to‐PA conduit replacement was 76% at 10 years. This is comparable to the reported 10‐year rate of freedom of conduit reoperations of other centers.^5,10,15^ In the present study, risk factors on univariate analysis for any reoperation included younger age at surgery, lower operative weight, and smaller implanted conduit diameter. This is consistent with Clark et al^10^ who reported that patient age and homograft size to influence the longevity of the pulmonary homograft. The findings of smaller homograft size and younger age at operation as risk factors for reoperation are apparent in other pediatric patients undergoing RV‐to‐PA conduit surgery.^28^

At the RCH, the current policy is to delay the Ross procedure until the child is past infancy, if feasible. If the patient requires surgery during infancy, the preference is to perform an initial surgical aortic valve repair.^29^ Due to excellent results with aortic valve repair, we tend to reserve Ross operation as the second line of management. The number of aortic valve repairs increased as compared to Ross operations during the recent years.^29–30^ Only patients with small aortic annulus would have Ross procedure without contemplation for an aortic valve repair as initial operation.^29–30^ Thus, postponing the Ross operation until later in childhood or, ideally, into adulthood may minimize both mortality and overall reoperation rates.

### Limitations

Our study is retrospective in nature and is limited by its design. Over the study period, surgical and perioperative techniques varied between patients. Although the groups were retrospectively matched, the selection of patients who underwent PDS banding was not prospectively randomized. Dimensions of the neoaortic root were not available in all patients.

## Conclusion

The Ross procedure in children can be performed with acceptable results. Children younger than 1 year of age have higher mortality, but not an increased autograft reoperation rate. Stabilization of the ST‐junction may reduce AI. The majority of patients are asymptomatic at last follow‐up. A smaller homograft inserted at the Ross procedure is a significant predictor for reoperation.
